# Tissue-independent and tissue-specific patterns of DNA methylation alteration in cancer

**DOI:** 10.1186/s13072-016-0058-4

**Published:** 2016-03-08

**Authors:** Yuting Chen, Charles E. Breeze, Shao Zhen, Stephan Beck, Andrew E. Teschendorff

**Affiliations:** CAS Key Laboratory of Computational Biology, CAS-MPG Partner Institute for Computational Biology, Shanghai Institute for Biological Sciences, Chinese Academy of Sciences, Shanghai, 200031 China; University of Chinese Academy of Sciences, 19A Yuquan Road, Beijing, 100049 People’s Republic of China; Medical Genomics, Paul O’Gorman Building, UCL Cancer Institute, University College London, 72 Huntley Street, London, WC1E 6BT UK; Statistical Cancer Genomics, Paul O’Gorman Building, UCL Cancer Institute, University College London, 72 Huntley Street, London, WC1E 6BT UK; Department of Women’s Cancer, University College London, 74 Huntley Street, London, WCIE 6AU UK

**Keywords:** DNA methylation, Cancer, Histone, Bivalency, Chromatin

## Abstract

**Background:**

There is growing evidence that DNA methylation alterations contribute to carcinogenesis. While cancer tissue exhibits widespread DNA methylation changes, the proportion of tissue-specific versus tissue-independent DNA methylation alterations in cancer is unclear. In addition, it is unknown which factors determine the patterns of aberrant DNA methylation in cancer.

**Results:**

Using HumanMethylation450 BeadChips (450k), we here analyze genome-wide DNA methylation patterns of ten types of fetal tissue, in addition to matched normal-cancer data for corresponding tissue types, encompassing over 3000 samples. We demonstrate that the level of aberrant cancer DNA methylation in gene promoters and gene bodies is highly correlated between cancer types. We estimate that up to 60 % of the DNA methylation variation in a cancer genome of a given tissue type is explained by the corresponding variation in a cancer genome of another type, implying that much of the cancer DNA methylation landscape is tissue independent. We further show that histone marks in normal cells are better predictors of aberrant cancer DNA methylation than the corresponding signals in human embryonic stem cells. We build predictors of cancer DNA methylation patterns and show that although inclusion of three histone marks (H3K4me3, H3K27me3 and H3K36me3) improves model accuracy, the bivalent marks are the most predictive. Finally, we show that chromatin accessibility of gene promoters in normal tissue dictates the promoter’s propensity to acquire aberrant DNA methylation in cancer in so far as it determines its level of DNA methylation in normal tissue.

**Conclusions:**

Our data show that a considerable fraction of the aberrant cancer DNA methylation landscape results from a mechanism that is largely tissue specific. Histone marks as specified in the normal cell of origin provide highly predictive models of aberrant cancer DNA methylation and outperform those derived from the same marks in hESCs.

**Electronic supplementary material:**

The online version of this article (doi:10.1186/s13072-016-0058-4) contains supplementary material, which is available to authorized users.

## Background

Aberrant DNA methylation (DNAm) is a well-established cancer hallmark [1–4]. Characteristic features of the cancer epigenome include promoter hypermethylation [4] and large mega-base scale blocks of hypomethylation [5–8], which often coincide with lamina-associated domains (LADs) [9] and domains of heterochromatin (H3K9me2 and H3K9me3) termed LOCKs [10]. Cancer-associated hypermethylation of gene promoters has been shown to be more frequent at genes that are bivalently or PRC2 marked in human embryonic stem cells (hESCs) [11–13], and this appears to be a universal signature across all types of cancer. Likewise, large hypomethylated blocks have been shown to be a universal feature of solid [8] and blood cancers [7]. Interestingly, hypermethylation at bivalently or PRC2-marked gene promoters, and large-scale block hypomethylation are also characteristic features of the DNAm landscape of aged normal tissue [14, 15]. Given that age is a major risk factor for many cancer types, it is plausible that age-associated epigenetic changes in normal cells contribute to a cell’s predisposition to undergo neoplastic transformation [1], with the transformation itself further aggravating these epigenetic changes [15].

Although many of the features of the cancer DNAm landscape appear to be universally valid across different cancer types, few studies have systematically explored the tissue specificity, or non-specificity, of these features at the level of individual genomic loci. For instance, although an interesting recent study by Nejman et al. [16] has shown that each CpG island (CGI) has an inherent tissue-independent propensity to become de novo methylated in cancer, this phenomenon has only been demonstrated across a few tissue types. Likewise, the demonstration that histone marks, specifically H3K27me3 and H3K4me3, in normal cells yield improved predictors of CGI hypermethylation in the corresponding cancer type, compared with the corresponding marks in hESCs, has only been demonstrated for colon tissue. Other studies have explored patterns of cancer DNAm in relation to gene expression patterns in the corresponding normal tissue, but were not highly quantitative, did not consider histone modifications and only used DNAm data from the older Illumina 27k technology [17, 18]. Further, motivated by a previous study, which has shown that the somatic mutational landscape of a given cancer type can be best predicted using histone marks in the corresponding normal cell type [19], we here decided to conduct a similar analysis in the context of predicting the cancer DNAm landscape, using several tissue types, as well using as the most recent histone modification data from the NIH Epigenomics Roadmap and DNAm data generated using the more comprehensive Illumina 450k bead arrays.

## Results

### The propensity for promoter hypermethylation and gene-body hypomethylation is highly correlated across cancer types

In order to understand the molecular rules which determine aberrant de novo DNAm in cancer, we decided to follow the strategy of Nejman et al. [16] and to focus on a background set of genomic sites which exhibit constitutively normal DNAm levels in a ground state. In order to avoid confounding by age [15, 20, 21], we defined this ground state to be that of DNAm levels in fetal tissue. In contrast to Nejman et al., however, we used a more comprehensive DNAm data set profiling ten different fetal tissue types (stomach, heart, tongue, kidney, liver, brain, thymus, spleen, lung and adrenal) [22] genome-wide with Illumina Infinium 450k bead arrays [23], thus allowing us to define a more objective set of sites with constitutively normal DNAm levels (“Methods” section). We focused on three different types of genomic elements, including gene promoters (defined as the region 200 bp upstream of the transcription start site—TSS), CGIs and gene bodies, resulting in 9063 constitutively unmethylated CGIs (cu-CGIs), 8360 constitutively unmethylated gene promoters (cu-GPs) and 4059 constitutively methylated gene bodies (cm-GBs) across all fetal tissue types (“Methods” section, Additional file 1: Table S1). Confirming our definitions, we observed a strong correlation of our cu-CGIs with those defined by Nejman et al. [16] using Agilent promoter DNAm arrays (Additional file 2: Table S2).

**Fig. 1:**
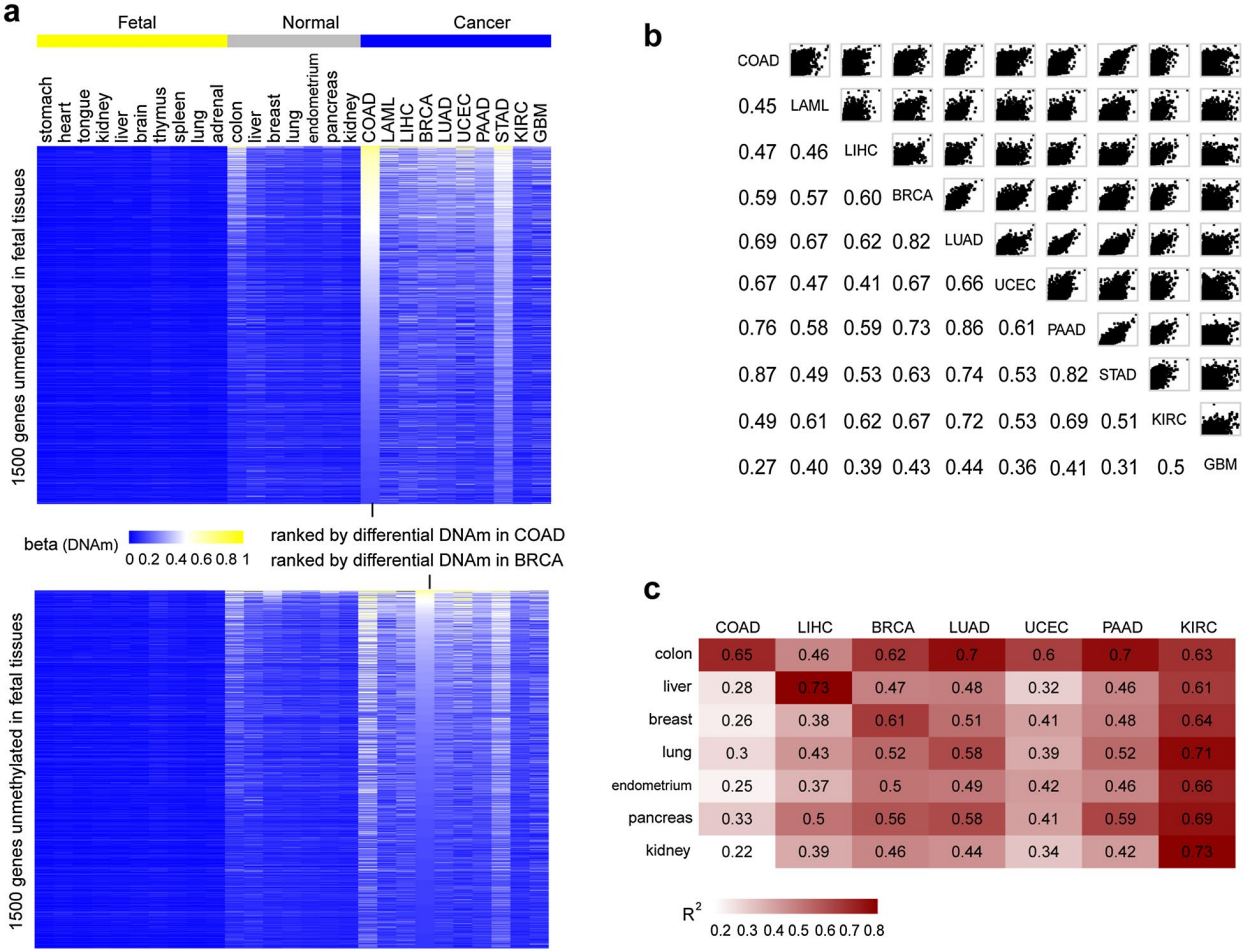
Tissue-independent cancer DNA methylation patterns. **a** Top heatmap depicts the DNA methylation values of 1500 top-ranked cu-GPs, ranked by level of hypermethylation in colon cancer (COAD), across all fetal tissue types, adult normal tissue and age-matched cancer types from the TCGA. Lower heatmap is the analog for top 1500 cu-GPs, ranked according to hypermethylation in breast cancer (BRCA). In every case, we show the average DNAm values in each phenotype. **b** *Upper diagonal* *Scatterplots* of average DNAm levels of the 8360 cu-GPs between each cancer type. *Lower diagonal* corresponding *R* ^2^ (Pearson) correlation values. **c** Heatmap of correlation *R* ^2^ values of the average DNAm levels of the 8360 cu-GPs in a given cancer type against the corresponding DNAm levels in normal tissue

In order to study the DNAm patterns of these genomic sites in cancer, we compared their DNAm levels in ten different TCGA cancer types (BRCA, COAD, KIRC, LIHC, LUAD, GBM, LAML, STAD, UCEC and PAAD, “Methods” section) to their respective normal tissues. For instance, focusing on colorectal adenoma carcinoma (COAD), we ranked the top 1500 cu-GPs in order of highest *β* values in colon cancer and compared their DNAm levels to those in other cancer types and age-matched normal tissues (Fig. 1a). Extending the results of Nejman et al., we observed that cu-GPs exhibited similar propensities to becoming methylated in other cancer types. This was particularly evident for colon and stomach (STAD) cancer, two tissue types that are developmentally similar. To formally quantify these correlations, we calculated *R*^2^ values from Pearson's correlations of the DNAm levels over all 8630 cu-GPs and for all pairwise combinations of ten tumor types (Fig. 1b). Most *R*^2^ values were relatively high confirming strong correlative patterns. Interestingly, correlations were also high between a given cancer type and its age-matched normal tissue (Fig. 1a). For instance, in the case of colon cancer, the most highly methylated cu-GPs were generally also the ones exhibiting most methylation in the age-matched normal colon (Fig. 1a). If this analysis was repeated using another cancer type, say breast cancer (BRCA), we observed a very similar pattern (Fig. 1a). To quantify this, we asked whether correlation *R*^2^ values between a given cancer and its normal tissue were in general higher than between cancer and normal comparisons from different tissue types. We were able to confirm this with statistical significance (Fig. 1c, Additional file 2: Fig. S1).

All of the above results were replicated had we used the set of cu-CGIs or cm-GBs (Additional file 2: Figs. S2, S3). This further supports the view that specific gene-centric patterns of aberrant DNAm in cancer are largely independent of cancer type, with mean *R*^2^ values between cancer types of 0.57, 0.55 and 0.5 for GPs, CGIs and GBs, respectively. Although the *R*^2^ values were significantly higher for GPs compared with GBs (Additional file 2: Fig. S4), this could be driven by the fact that 450k probe density was also highest for GPs, with average DNAm values in these regions being estimated over probes that are more highly correlated (Additional file 2: Fig. S5).

### Aberrant cancer DNAm patterns are predicted best by bivalently marked histone signals measured in normal tissue

Having demonstrated that a substantial component of the aberrant DNAm landscape in cancer appears to be independent of tissue type, we next decided to explore the molecular determinants of tissue-specific cancer DNAm. Given that histone signals in normal tissue have recently been demonstrated to be good predictors of the tissue-specific somatic mutation [19, 24] and DNAm [16] landscape in cancer, we decided to investigate this more comprehensively in the context of DNAm.

**Fig. 2:**
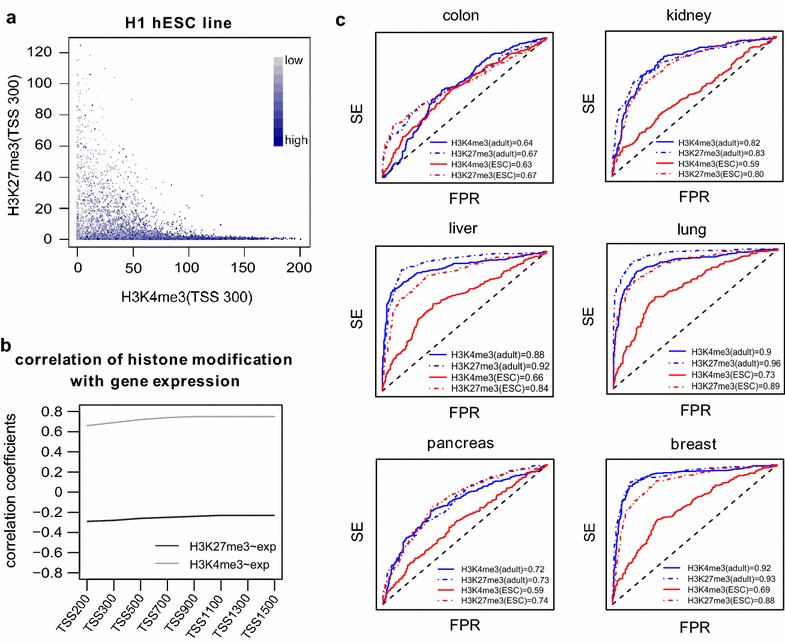
Prediction of tissue-specific cancer DNAm patterns from bivalent marks in normal cells. **a** *Scatter plot* shows the association between gene expression with promoter H3K4me3 (*x*-axis) and H3K27me3 (*y*-axis) occupancy. **b** Pearson's correlation coefficients calculated between promoter H3K4me3 and H3K27me3 modification with corresponding gene expression levels in H1 hESC line, as function of the window size (bp) centered at the TSS over which the signal is estimated. **c** ROC-AUC analysis assessing the predictive potential of cancer-associated hypermethylation at unmethylated GPs in normal tissue from H3K4me3 and H3K27me3 promoter signals in normal tissue and hESC cells, as indicated. *Each panel* is for a given tissue and cancer type

We downloaded histone signal data for three major marks (H3K4me3, H3K27me3 and H3K36me3) in normal tissue types for which corresponding TCGA DNAm data were available (“Methods” section). We defined histone signals over gene promoters using a procedure which tuned the window size around the gene promoter to optimize the correlation between H3K27me3/H3K4me3 histone signals and gene expression (RNA-Seq) for H1 hESC line (“Methods” section, Fig. 2a, b). We chose the H1 cell line because it exhibited a relatively high similarity of histone modification pattern with the majority of other human embryonic stem cell lines **(**“Methods” section, Additional file 2: Fig. S6). This procedure resulted in ±300 bp around the TSS as an optimal (or near optimal) window size for histone signals defined over gene promoters (Fig. 2b, Additional file 2: Fig. S7). We then used this window size to define corresponding histone signals in normal cell types. Specifically, focusing on the cu-GPs, we computed for each of the two histone marks H3K4me3 and H3K27me3 in the corresponding normal tissue, an AUC, assessing its ability to predict DNA hypermethylation at the same cu-GPs in the corresponding cancer (“Methods” section). In order to benchmark performance, we compared the AUCs to those obtained using the same histone marks in hESCs. We observed that H3K4me3 and H3K27me3 signals derived in the normal tissue of the same cell type were better predictors of cancer hypermethylation at cu-GPs than the corresponding signals as estimated in hESCs (paired Wilcoxon's test one-tailed *P* value = 0.0007, Fig. 2c, Additional file 2: Fig. S8). Interestingly, we observed that this improvement in prediction was much more marked for the H3K4me3 signal. In fact, while the hESCs H3K27me3 signal already yielded a relatively high prediction accuracy with only a marginal improvement seen for the normal-tissue H3K27me3 signal, the corresponding hESCs signal for H3K4me3 was generally not predictive, or only marginally so (Fig. 2c). In general, the H3K27me3 and H3K4me3 signals performed similarly in normal adult tissue (Fig. 2c). Thus, these results generalize the observations made previously in the case of colon tissue [16] to several other normal tissue types.

### H3K36me3 histone signal measured in normal tissue type outperforms the corresponding hESC signal as predictor of gene promoter hypermethylation and gene-body hypomethylation in cancer

**Fig. 3:**
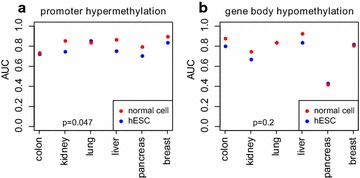
Prediction accuracy of tissue-specific cancer DNAm patterns from the H3K36me3 signal. **a** *Scatter plot* shows the area under the curve (AUC) prediction accuracy (*y*-axis) of promoter hypermethylation in cancer from the H3K36me3 signal in the corresponding normal tissue or hESC, as indicated. Normal/cancer tissues considered include colon (COAD), kidney (KIRC), lung (LUAD), liver (LIHC), pancreas (PAAD) and breast (BRCA). *P* value is from a paired Wilcoxon rank sum test. **b** As **a**, but now predicting gene-body hypomethylation in cancer

Next, we decided to extend the analysis described above to include other histone marks. We considered H3K36me3 for various reasons. First, this mark featured prominently as a predictor of somatic mutation frequency in cancer [19]. Second, we previously found a reader (WHSC1) and an editor (CBX7) of this mark to be among candidate key master regulators of the cancer DNA methylome [25]. Thus, we posited that this mark in normal cells may carry important predictive information of which genes are aberrantly methylated in cancer. In contrast to the bivalent marks, the H3K36me3 signal was estimated over the gene body, due to its role in transcription elongation (“Methods” section). We observed that the signal derived in normal tissues was more predictive of promoter hypermethylation in cancer than the corresponding signal in hESCs (one-tailed paired Wilcoxon's test *P* = 0.047, Fig. 3a). Although overall accuracies were high, comparison of H3K36me3 to the bivalent marks revealed marginally worse performance (Additional file 2: Fig. S9).

Since H3K36me3 is mainly distributed over the gene body, we also investigated whether the mark would better predict gene-body hypomethylation in cancer. For this analysis, we focused on the cm-GBs and asked how well the marks in normal cells would predict cancer-associated hypomethylation. We found that the H3K36me3 signal could better predict tumor-associated gene-body hypomethylation than the corresponding signal measured in hESCs for half of the six tissue types (Fig. 3b). Although there was no statistical significance, for those tissues exhibiting a larger difference in AUC, the AUC was always higher for the H3K36me3 mark in normal tissue (Fig. 3b). Interestingly, predicting cancer-associated gene-body hypomethylation with H3K4me3 and H3K27me3 promoter signals in normal tissues was also possible, although, overall, H3K36me3 performed marginally better than the bivalent marks (Additional file 2: Fig. S10).

### Multivariate histone signal models allow highly accurate prediction of cancer-associated hypermethylation

**Fig. 4:**
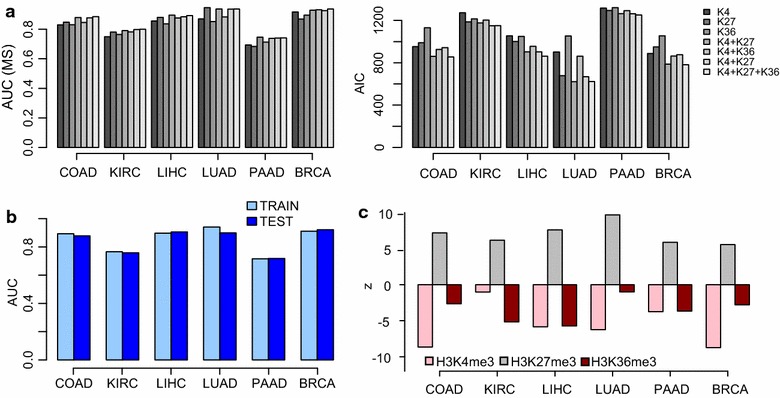
Prediction accuracy of tissue-specific cancer DNAm patterns from the H3K36me3 signal. **a** *Left panel* depicts a *barplot* of the AUC of seven different models (as obtained in the cross-validation model selection step) in predicting promoter DNA hypermethylation in cancer, for each of six different cancer types (COAD, KIRC, LIHC, LUAD, PAAD and BRCA), as indicated. *High AUC values* indicate better models. *Right panel* depicts the Akaike information criterion (AIC) values of each model in each cancer type. *Lower AIC values* indicate better models. **b** AUC values of optimal model in the mode selection (training) and blind test sets and for each cancer type. **c** For the model including all three histone signals as predictors, we plot the estimated *z*-statistics of each predictor of the trained model. Positive (negative) associations indicate that larger (lower) signal values correlate with increased promoter hypermethylation in cancer

To more formally compare the three histone signals to each other and to more objectively assess prediction performance, we used a 70 % training 30 % test set strategy whereby differentially hypermethylated and non-hypermethylated genes were assigned in equal proportions to each set (“Methods” section). We first used a forward selection strategy to train a total of seven nested models with all potential combinations of histone marks as predictors within a logistic regression model framework. We used an internal validation set to select a best predictive model from the training set for each tissue type, which was then finally evaluated in the blind test set (“Methods” section). In addition, all seven models were compared using the Akaike information criterion (AIC). Overall, across the six tissue types, both model selection procedures (forward selection and AIC) revealed that a three-predictor (histone) model performed best, typically achieving AUC values of over 0.8 (Fig. 4a). Importantly, performance in the training and test sets was similar, although marked variation across tissue types was evident (Fig. 4b). Of note, the three-predictor model yielded highly consistent predictive patterns across the six tissue types, with H3K27me3 emerging overall as the top predictor, correlating positively with promoter cancer hypermethylation, whereas H3K4me3 and H3K36me3 signals correlated negatively (Fig. 4c). This indicates that higher levels of promoter H3K4me3 or gene-body H3K36me3 in normal tissue is protective of cancer-associated promoter hypermethylation.

### Patterns of cancer DNA methylation across DNase hypersensitive sites

**Fig. 5:**
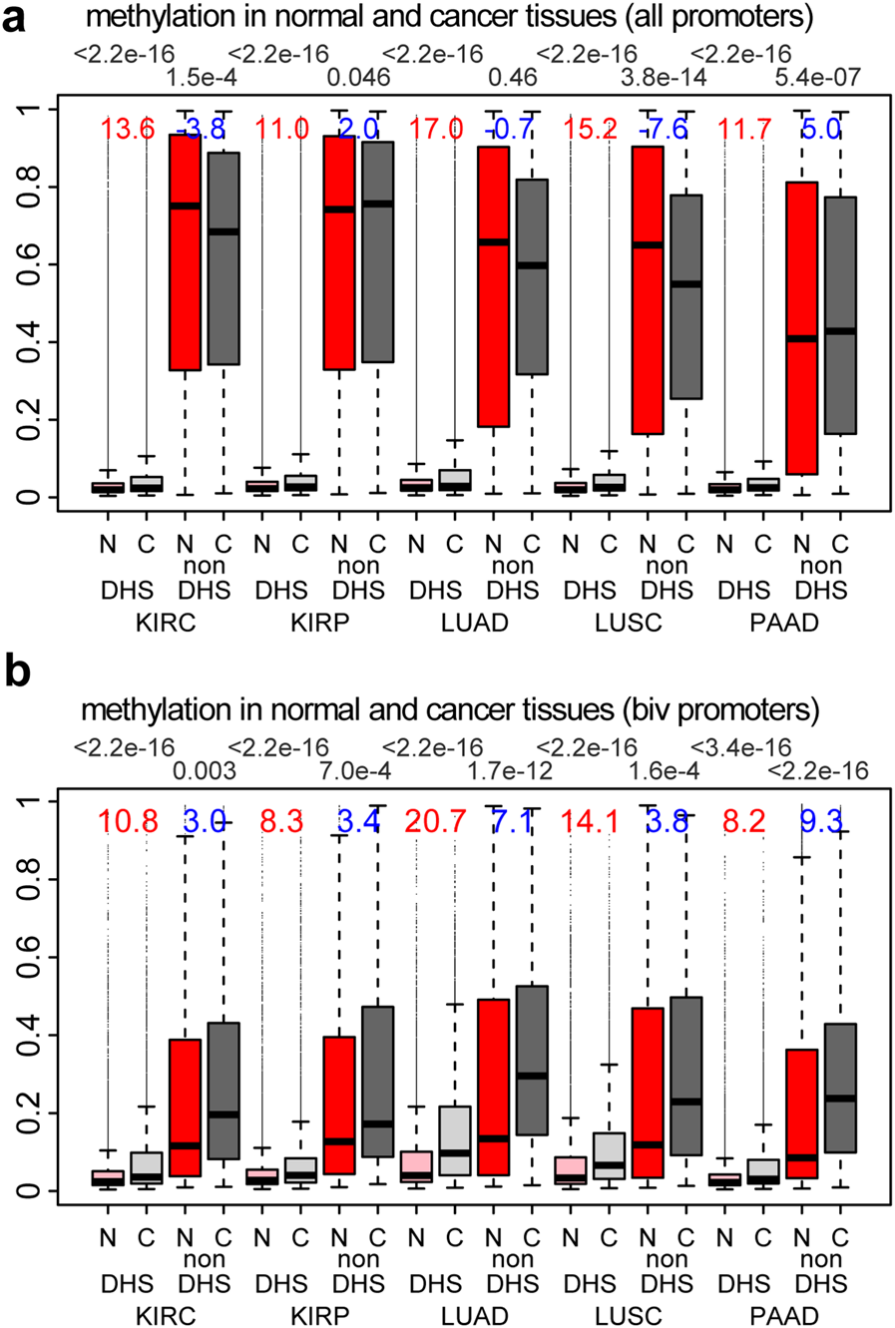
Patterns of promoter DNAm change in cancer depending on open/closed chromatin. **a** *Boxplots* of DNAm *β* values of gene promoters, stratified according to normal/cancer tissue and whether in or outside of a DHS region, where DHS status is determined in the corresponding normal cell type. DHS data were available for three normal tissues (lung, kidney and pancreas), and hence, there were a total of five cancer types (KIRC, KIRP, LUAD, LUSC and PAAD). *Above boxplots*, we give the *t* statistics between normal (*N*) and cancer (*C*). *Red* labels the *t* statistics when restricted to DHS regions, and *blue* labels *t* statistics when restricted to non-DHS regions. *Above the plot* we give the corresponding *t* test *P* values. **b** As **a**, but now for bivalently marked gene promoters, with bivalency as determined in the normal cell type

Finally, we decided to investigate how the patterns of aberrant cancer methylation may depend on the accessibility of chromatin in normal tissue. To this end, we obtained DNase hypersensitive sites (DHSs), as determined by the NIH Epigenomics Roadmap [26, 27] for a number of primary (adult) and fetal cell types, which included lung, kidney and pancreas (“Methods” section). First, we considered all gene promoters, regardless of their DNAm levels in normal tissue, and asked whether promoters in DHSs are more likely to undergo cancer hypermethylation than promoters located in closed chromatin regions. Across the three tissue types (lung, kidney, pancreas) and considering in total five cancer types (LUAD, LUSC, KIRC, KIRP and PAAD), in every single case we observed a stronger differential methylation pattern for promoters in DHSs, possibly owing to their lower level of DNAm in normal tissue (Fig. 5a). GPs outside DHSs were generally highly methylated in normal tissue and did not exhibit a clear trend toward either hyper- or hypomethylation in cancer (Fig. 5a). Next, we repeated the same analysis but now restricting to tissue-specific bivalent GPs. The bivalent GPs located outside DHSs exhibited significantly lower levels of DNAm in normal tissue compared with non-bivalent GPs, resulting in a significant trend toward hypermethylation in cancer, although not as significant as for bivalent GPs located in DHSs (Fig. 5b). A similar finding was evident by restricting to the class of cu-GPs identified earlier (Additional file 2: Fig. S11). Together these data suggest that chromatin accessibility of GPs in normal tissue only determines the propensity of cancer hypermethylation in so far as it determines the level of DNAm in the normal tissue.

## Discussion

The results presented here confirm those of Nejman et al. [16] and Sproul et al. [17], while also extending their key findings to several other tissue types and gene regions. Specifically, we have here shown that the aberrant DNAm landscape of any given cancer type is highly correlated (*R*^2^ values >0.5) across cancer types, a result which was valid not only for CGIs but also for gene promoters and gene bodies. As proposed by Nejman et al. for the case of CGIs, this suggests that each gene-associated region, be it a promoter or gene body, has an intrinsic propensity to acquire aberrant DNAm in cancer.

Our second main contribution is the demonstration that bivalent histone marks at gene promoters, as defined in normal cells, are a better predictor of promoter hypermethylation in cancer compared with the corresponding marks in hESCs. This result was demonstrated across six different tissue types, while Nejman et al. only considered colon tissue. In addition, we also considered the H3K36me3 mark in normal cell types, which in general also resulted in better predictions of aberrant cancer DNAm compared with the signal as defined in hESCs. Thus, we built prediction models using all three histone marks (H3K27me3, H3K4me3 and H3K26me3), which demonstrated that each one of these marks adds predictive value, although the bivalent marks were generally the most predictive of promoter hypermethylation in cancer. Indeed, our data further support the view that H3K27me3 in normal cells is the most predictive mark of promoter cancer hypermethylation, followed closely by H3K4me3, which acts to protect the promoters from cancer hypermethylation, consistent with the findings of Sproul et al. [17] who only considered gene expression levels. This indicates that promoters marked only by H3K27me3 in normal cells are the most likely to undergo hypermethylation in cancer, with bivalently marked promoters showing marginally less propensity to do so. Interestingly, our data further point to the H3K4me3 promoter mark in normal tissue as being particularly informative of which promoters do not undergo hypermethylation in cancer, with the corresponding mark in hESCs not being predictive. It will be interesting to explore these results further in the context of a recent study showing that promoters characterized by broad H3K4me3 peaks in normal cells may mark candidate tumor suppressor genes [26].

Finally, we have also explored the cancer DNAm landscape in relation to open/closed chromatin states in the corresponding normal cell types. Not unexpectedly, we observed a very strong inverse correlation between promoter DNAm levels and chromatin accessibility, both defined in the same normal tissue. As a result of this, promoters in closed chromatin regions, which are generally methylated, did not exhibit a consistent and significant trend toward aberrant DNAm in cancer. In contrast, promoters in open chromatin regions were invariably unmethylated in normal tissue and generally exhibited hypermethylation in cancer. Interestingly, however, this pattern changed slightly when we focused on bivalent promoters or promoters which are unmethylated in a normal tissue type, since these promoters, even those outside of DHSs, generally exhibited significant hypermethylation in cancer. Thus, irrespective of chromatin accessibility, the propensity of a promoter region to undergo DNAm changes in a specific cancer type is mainly determined by its level of DNAm in the corresponding normal tissue.

## Conclusions

In summary, this work shows that much of the cancer DNAm landscape is determined by a mechanism which is largely independent of the original tissue type. Nevertheless, bivalent histone marks in normal cells are better predictors of aberrant cancer DNAm patterns than the corresponding marks defined in hESCs.

## Methods

### Data collection

DNAm data of 37 fetal samples were downloaded from the Stem Cell Matrix Compendium version 2 (SCM2) [22]. There were ten types of fetal tissues in total, including stomach, heart, tongue, kidney, liver, brain, thymus, spleen, lung and adrenal gland. DNAm data of normal/cancer tissues were downloaded from The Cancer Genome Atlas (TCGA) Data Portal and included colon, kidney, liver, lung, pancreas, stomach, skin, brain, endometrium and breast tissue.

All DNAm data were generated on the Illumina Infinium HumanMethylation450 BeadChip, which provides genome-wide coverage of 99 % RefSeq genes and 96 % CGIs. Probes on this bead array are distributed across all gene regions (promoter region, 5′ UTR, 1st exon, gene body and 3′ UTR), and all CGI regions, including shores and shelves. Such a broad coverage of genes and CGIs make it possible for us to calculate *β* values for specific gene regions and CGIs.

ChIP-Seq data for H3K4me3, H3K27me3 marks measured in normal colon mucosa, normal liver, normal lung, normal pancreas and normal breast, as well as all H3K36me3 marks were downloaded from the NIH Roadmap Epigenomics Mapping Consortium Web site (http://www.roadmapepigenomics.org/). The H3K4me3 and H3K27me3 signal data for kidney were downloaded from the International Human Epigenome Consortium (IHEC) Web site (http://ihec-epigenomes.org/).

Gene expression data (RNA-Seq) for the human embryonic stem cell line H1 were downloaded from the NIH Roadmap Epigenomics Mapping Consortium Web site.

### Processing of TCGA DNA methylation data

Level-3 TCGA data were further processed as follows: Probes with a coverage of <70 % were removed. Missing values of remaining probes were estimated using the impute.knn function from the impute package (*k* = 5) [28]. BMIQ was then applied to adjust for the type II probe bias [29]. To assess potential confounding by technical sources of inter-sample variation or unknown batch effects, we applied singular value decomposition (SVD) to each TCGA data set to check that the top component of variation of the data is associated with normal/cancer status, an approach we have validated previously [30]. After that, *β* values of probes located within 200 base pairs upstream of the TSS of a gene or, alternatively, located within a gene body (excluding the 1st exon) were averaged for each gene, yielding separate promoter and gene-body DNAm values for each gene. We also calculated an averaged *β* value for each promoter CGI.

### Definition of constitutively unmethylated gene promoters, CGIs and constitutively methylated gene bodies

We defined sets of constitutively unmethylated (cu) gene promoters and CGIs across all ten types of fetal tissue. To declare a methylation value as being unmethylated in a given sample, we inferred sample-specific thresholds from the application of BMIQ, which assigns all methylation values to three states (unmethylated, half-methylated, fully methylated) according to a three-state beta-mixture model [29]. Specifically, if the gene promoter or CGI methylation value was less than the lower threshold inferred using BMIQ for that particular sample, that value was declared unmethylated. In the case of gene bodies, we used the upper threshold of BMIQ to determine whether the average gene-body methylation was “methylated.” Specifically, we declared values larger than the upper threshold as methylated.

### Processing of ChIP-Seq data

All ChIP-Seq data were downloaded in bigWig format from the Web sites mentioned earlier. First, we converted bigWig formatted data to bedGraph format using the function bigWigToBedGraph provided by the UCSC Genome Bioinformatics Web site. The bedGraph formatted data could be directly read into R. Each row of a bedGraph file gives the start and end position of each tag and an associated minus log10 *p* value, *S* = −log_10_[*P*] for this region. All of these data were of the hg19 genome assembly, so we used the gene annotation file of the same assembly to map tags in bedGraph files to gene promoter or gene-body regions (gene body defined here from the 1st exon to the last one, including introns). An averaged $\left\langle S \right\rangle$ signal value was assigned to each gene by averaging *S* values from the matched tags.

In the case of gene promoters, the window size for promoter H3K4me3 and H3K27me3 signals needs to be carefully chosen. To tune this parameter, we first calculated average H3K4me3 and H3K27me3 signal values for each gene promoter using different window sizes (±200, 300, 500, 700, 900, 1100, 1300 and 1500 bp from the gene TSS). Then, we assessed the correlation (Pearson's correlation coefficients) of the resulting histone signals (calculated for different window sizes) with gene expression, all measured in H1 hESC cell line.

The H1 cell line was selected from a total of eight different human embryonic stem cell lines (WA7, I3, H1, H9, UCSF4, HUES6, HUES48 and HUES64). The selection was done by comparing the similarity between gene promoter (TSS300) H3K4me3/H3K27me3 and gene body (from the end of the 1st exon to the last one, excluded introns) H3K36me3 modifications. We used pairwise Pearson's correlation coefficients between the eight hESC lines as a measure of similarity. The H1 cell line exhibited the hightest similarity to most other hESC lines.

### Processing of H1 mRNA expression data

We downloaded the RNA-Seq gene expression data (quantified as RPKM) of the H1 human embryonic stem cell line from NIH Roadmap Epigenomics Mapping Consortium Web site. We substituted zero values with the smallest positive value. As a threshold for calling expressed and non-expressed genes, we used ten reads. Since the total number of reads used in the generation of the data is around 39 million [26] and since the average length of genes in the whole genome is about 1 kb, we used a rounded value of −2 $\left( {\log_{2} \left( {\frac{10}{39 \times 1}} \right)\sim - 1.96} \right)$ as a threshold for calling a gene expressed or not. In other words, if a gene has <10 matched reads (log_2_[RPKM] < −2), we assign a log_2_[RPKM] value of −2 to it.

### Differential DNA methylation analysis

Two tailed t-tests were performed for each gene separately to detect differential promoter, or gene-body, DNAm in tumors compared with normal samples. We ranked genes by *t* statistic, with the resulting top N genes declared to be differentially methylated, while the bottom N genes were defined as not differentially methylated. *N* is a varying number used to assess the stability of our method. Because gene promoter and CGI levels are usually unmethylated in normal tissue, in these cases we ranked genes by the level of hypermethylation (i.e., by positive *t* statistics) in cancer compared with normal. Correspondingly, for gene-body DNAm, we ranked genes according to the negative *t* statistic, ranking at the top those genes exhibiting the largest decreases in gene-body methylation in cancer.

### Histone mark prediction of cancer DNA methylation patterns

We used a varying threshold to define the histone mark occupancy (signal value) as a binary state at individual genes. Since each gene is also defined as differentially methylated in cancer or not (depending also on the parameter *N* of top-ranked genes), a two-by-two contingency table for each histone modification threshold was generated, and a ROC curve was plotted out with the sensitivity and one-specificity calculated from the table. From this, we then estimated the area under the curve (AUC), indicating the prediction accuracy of each histone mark. A value of AUC was obtained for different choices of *N*. For all three histone marks (H3K4me3, H3K27me3 and H3K36me3), we chose *N* of 300 as this threshold gave us a consistent and robust result. For a given same value of *N*, we compared the prediction accuracy for each histone mark measured in normal tissue to the prediction accuracy of the corresponding marks measured in hESCs. Paired Wilcoxon's tests across all normal tissue types were performed to assess whether the difference between the AUCs derived from normal tissue and hESCs was statistically significant. Robustness of results to variations in *N* was assessed by comparing AUC values for different values of *N*.

### Prediction of promoter DNA hypermethylation in cancer using multivariate histone signal models

In order to assess the interplay between histone signals in predicting promoter DNA hypermethylation in cancer, we considered multivariate logistic regression models. Differentially hypermethylated and not differentially hypermethylated gene promoters in each cancer type were defined as the top 1000 and bottom 1000 genes (totally 2000), ranked by their *t* statistic. We then used a number of different model selection strategies to identify the best predictive logistic regression model among all models representing different possible combinations of one, two or three histone marks (seven models in total: 1 × 3, 3 × 2, 3 × 1). In one approach, we compared the seven models using the estimated AIC values (smaller AIC values indicate better models). In an alternative approach, we split the 2000 genes into a 50 % training set, a 20 % internal validation set (used for model selection) and a 30 % true blind test set, in equal proportions. The strategy here was to use the training set to learn the seven different logistic regression models and to then use an internal cross-validation (or model selection) step to evaluate each of the learned models in the blinded 20 % internal validation set, allowing us to assess which of the trained predictive models generalized best in a blind validation set. Finally, in order to check that our model selection has not introduced overfitting, the selected best model was tested using the true blind test set (30 %). Performance evaluation of all models in all three sets (training, internal test and true blinded) was assessed by computing the AUC.

### Correlation analysis of DNAm patterns

For each background set of cu-GPs, cu-CGIs or cm-GBs, we calculated the Pearson correlation coefficients of their average DNAm values in each cancer type with the corresponding values in their respective normal tissue. *R*^2^ values between cancer and normal tissue of the same type were compared (using a *t* test) to those of cancer-normal comparisons of different tissue types. We also used Pearson's correlations to calculate the correlation of DNAm patterns at cu-GPs, cu-CGIs and cm-GBs, between cancer types.

### 450k probe density for each genomic region

Probe density was calculated by dividing the number of 450k probes in a given genomic region by the length of the region. After having mapped the Illumina 450k probes annotated as “TSS200” or “Body” to all genes in the genome, we divided the number of probes mapped to the same gene by the distance between two matched probes which are farthest from each other. The density of probes over CGIs was calculated in a similar way. After mapping, for each CGI we counted the number of matched probes and the length of the CGI (the end site − the start site). Then, we did pairwise comparison of difference in probe density over the three genomic types of region by *t* test.

## Additional files

10.1186/s13072-016-0058-4 Excel Table Spreadsheet listing details of the constitutively unmethylated CGIs (cu-CGIs), constitutively unmethylated gene promoters (cu-GPs) and constitutively methylated gene bodies (cm-GBs).
10.1186/s13072-016-0058-4 Pdf document containing all Supplementary Figures S1–11, as well as Supplementary Table S2.

## Abbreviations

DNAm: DNA methylation
hESC: human embryonic stem cell
DHS: DNase hypersensitive site
RPKM: reads per kilobase of exon per million reads
TSS: transcription start site
CGI: CpG island
