# Supplementary material for: Tissue-independent and tissue-specific patterns of DNA methylation alteration in cancer
Source: Epigenetics Chromatin. 2016 Mar 8;9:10. doi: 10.1186/s13072-016-0058-4 (PMC4782576; doi:10.1186/s13072-016-0058-4)
Supplement: Supplementary file 2 — 10.1186/s13072-016-0058-4 Pdf document containing all Supplementary Figures S1–11, as well as Supplementary Table S2. [file 13072_2016_58_MOESM2_ESM.pdf]

Supplementary Information for:

Tissue-independent and tissue-specific patterns of DNA methylation alteration in cancer

Yuting Chen [1,2], Charles Breeze [3], Shao Zhen [1], Stephan Beck [3], Andrew E. Teschendorff [1,4,5*]

Corresponding author: Andrew E. Teschendorff- a.teschendorff@ucl.ac.uk

1. CAS Key Lab of Computational Biology, CAS-MPG Partner Institute for Computational Biology, Shanghai Institute for Biological Sciences, Chinese Academy of Sciences, Shanghai 20031, China.
2. University of Chinese Academy of Sciences, 19A Yuquan Road, Beijing 100049, P. R. China.
3. Medical Genomics, Paul O'Gorman Building, UCL Cancer Institute, University College London, 72 Huntley Street, London WC1E 6BT, United Kingdom.
4. Statistical Cancer Genomics, Paul O'Gorman Building, UCL Cancer Institute, University College London, 72 Huntley Street, London WC1E 6BT, United Kingdom.
5. Department of Women's Cancer, 74 Huntley Street, University College London, London WCIE 6AU, United Kingdom.

Contents

Supplementary Figures: S1 – S11

Supplementary Table S2

SUPPLEMENTARY FIGURES:

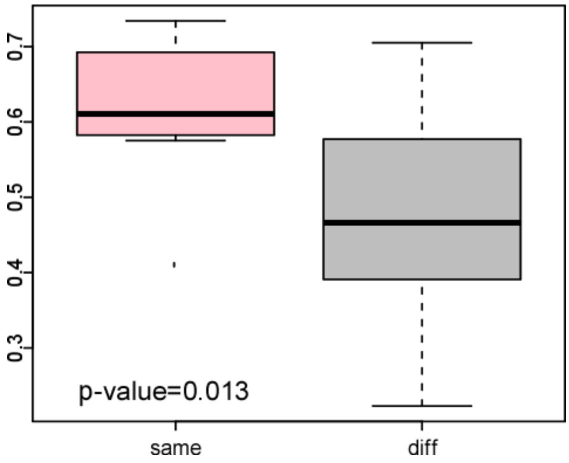

Supplementary Figure S1. Cancer-normal correlation R^2 values for cu-GPs.

Box plots comparing the R^2 values (y-axis) of the average DNAm levels of the 8360 cu-GPs hypermethylated in a given cancer type against the corresponding DNAm levels in normal tissues of the same or different tissue type. (one-sided P-value = 0.013; t-test).

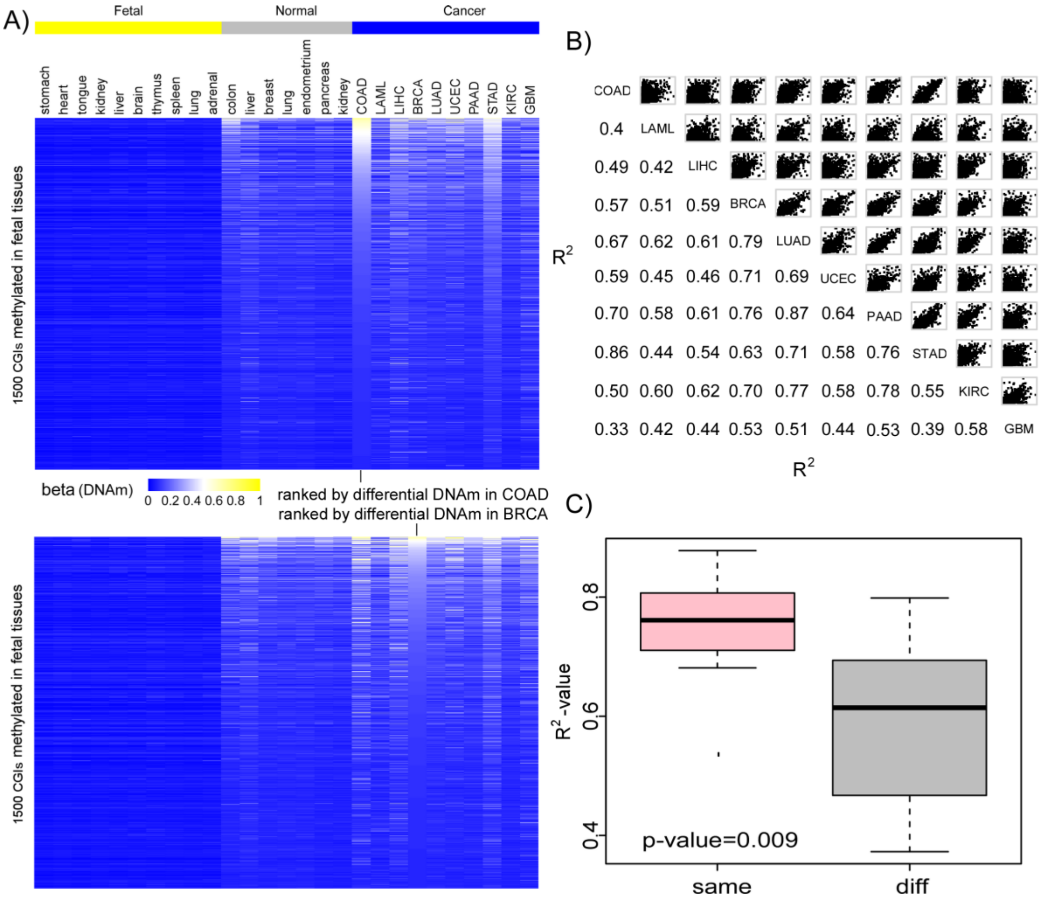

Supplementary Figure S2. Tissue-independent CGI methylation patterns in cancer. A) Top heatmap depicts the DNA methylation values of the top 1500 cu-CGIs, ranked by level of hypermethylation in colon cancer (COAD), across all fetal tissue types, adult normal tissue and age-matched cancer-types from the TCGA. Lower heatmap is the analogue for the case of the top 1500 cu-CGIs ranked according to hypermethylation in breast cancer (BRCA). In every case we show the average DNAm values in each phenotype. **B)** Upper diagonal: scatterplot of average DNAm levels for the 8624 cu-CGIs shown in the top panel of A) in each cancer type against each other. Lower diagonal: corresponding R^2 (Pearson) correlation values. **C)** Box plot showing the difference between the R^2 values (Pearson) of the average DNAm levels of the 8624 cu-CGIs hypermethylated in a given cancer type against the corresponding DNAm levels in normal tissue of the same or different tissue type. (one-sided P-value = 0.009; t-test).

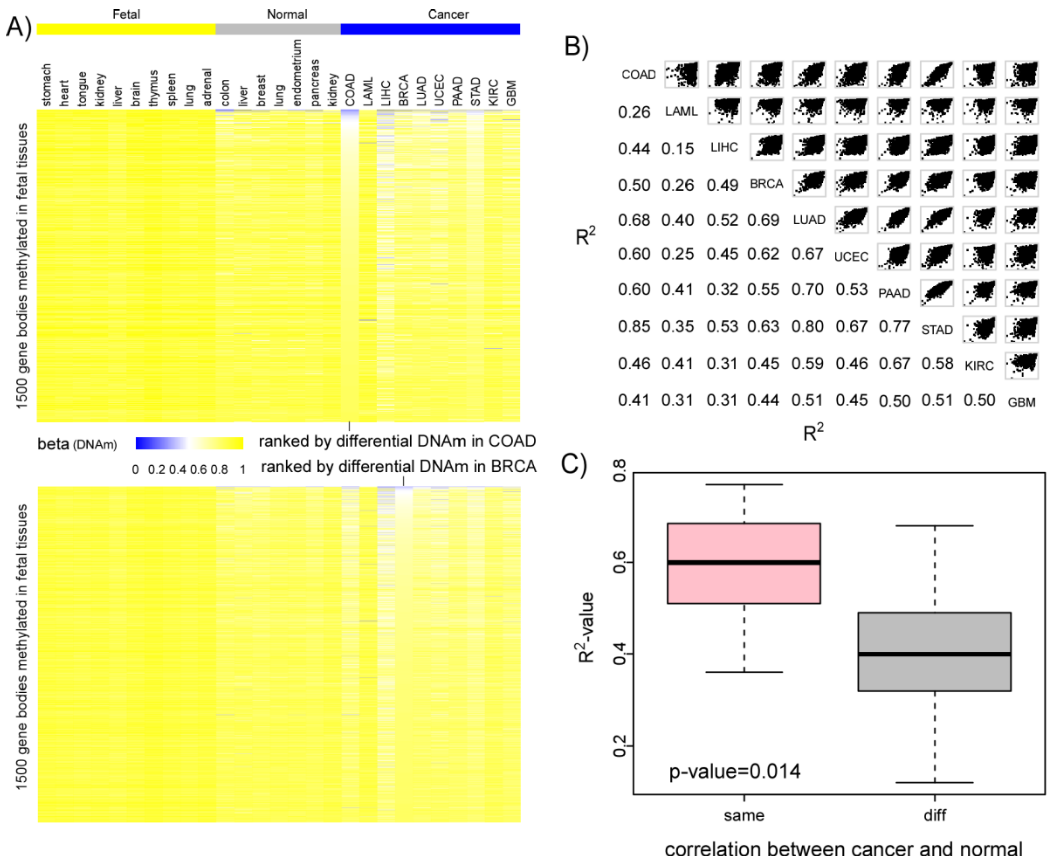

Supplementary Figure S3. Tissue-independent gene body DNA methylation patterns in cancer. A) Top heatmap depicts the DNA methylation values of the top 1500 cm-GBs, ranked by level of hypomethylation in colon cancer (COAD), across all fetal tissue types, adult normal tissue and age-matched cancer-types from the TCGA. Lower heatmap is the analogue for the case of the top 1500 cm-GBs ranked according to hypomethylation in breast cancer (BRCA). In every case we show the average DNAm values in each phenotype. **B)** Upper diagonal: scatterplot of average DNAm levels for the 4059 cm-GBs shown in the top panel of A) in each cancer type against each other. Lower diagonal: corresponding R^2 (pearson) correlation values. **C)** Box plot showing the difference between the R^2 values (Pearson) of the average DNAm levels of the 4059 cm-GBs hypomethylated in a given cancer type against the corresponding DNAm levels in normal tissues of the same or different tissue type (one-sided P-value = 0.014; t-test).

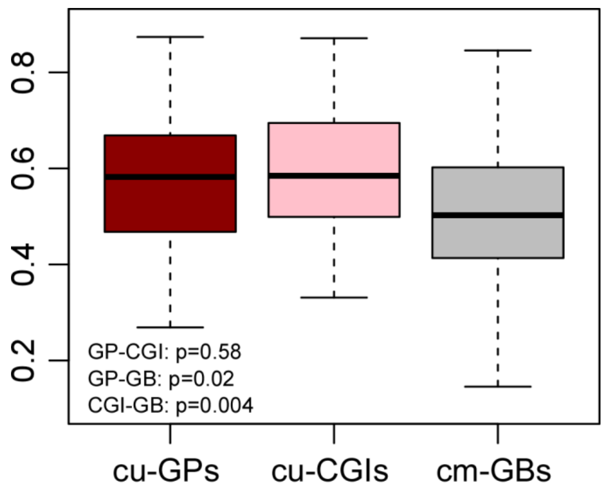

Supplementary Figure S4. Comparison of R^2 values between cancer types for cu-GPs, cu-CGIs and cm-GBs. Y-axis shows the correlation R^2 values calculated between 7 cancer types using Pearson correlation for three genomic elements: cu-GPs, cu-CGIs and cm-GBs. R^2 values were compared by pairwise Wilcoxon test (paired) between cu-GPs and cu-CGIs, cu-GPs and cm-GBs, cu-CGIs and cm-GBs. Difference of median R^2 values between cu-GPs and cm-GBs were significant with P-value equaling 0.02.

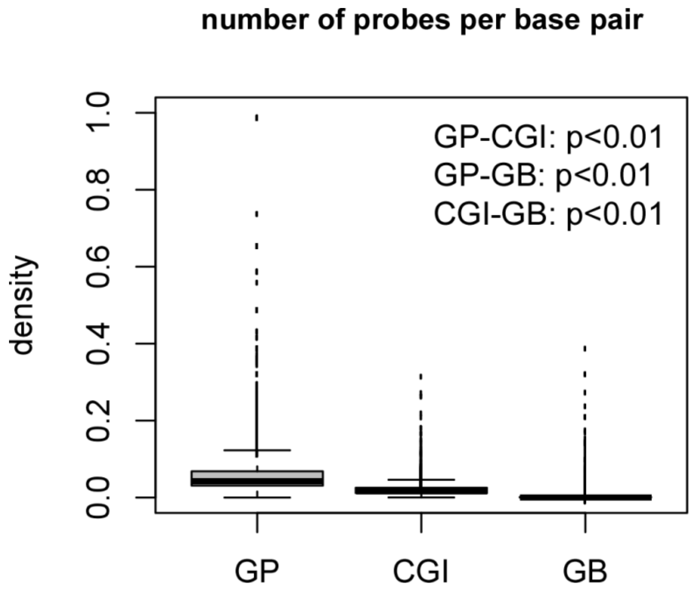

Supplementary Figure S5. Probe density for three genomic regions. Boxplot shows the probe density of three genomic elements (gene promoter, CGI and gene body) for all genes/ CGIs. We compared the density of probes between different genomic regions using pairwise t-tests. All three tests gave very significant p-values denoting that gene promoter has the highest probe density.

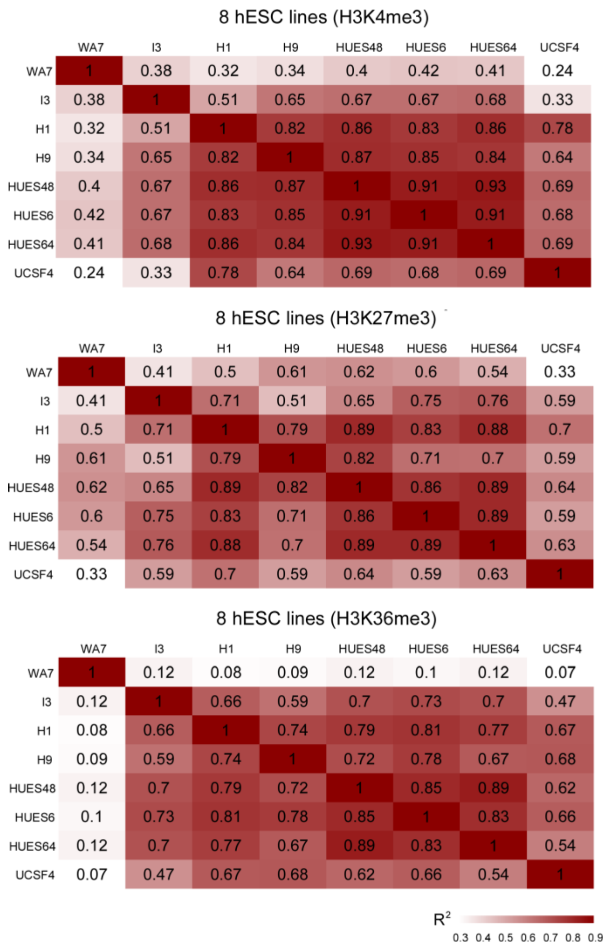

Supplementary Figure S6. Selection of H1 as a representative hESC line. Promoter H3K4me3 and H3K27me3 signal values were averaged over a -/+ 300bp window around TSS for every gene, while H3K36me3 signal values were averaged over gene body regions (from the end of the 1st exon to the last one, excluding introns). Pairwise Pearson correlation coefficients were calculated and R^2 values were shown in the three heat maps.

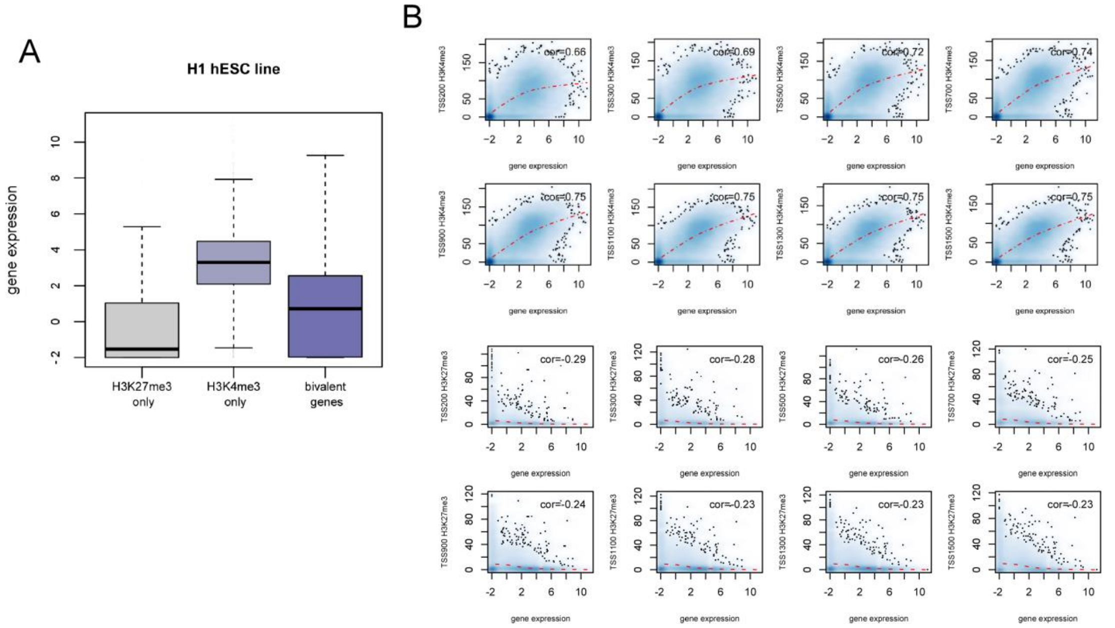

Supplementary Figure S7. Selection of the best window size for promoter H3K4me3 and H3K27me3 signal. A) Boxplots showing the expression level of genes only marked by H3K27me3/H3K4me3 modification or both. **B)** The H3K4me3 and H3K27me3 signal value over gene promoters were calculated around the transcription start sites (TSSs) using several window sizes: 200bp, 300bp, 500bp, 700bp, 900bp, 1100bp, 1300bp, 1500bp. Scatter plots show the correlation between histone signals for different window sizes and gene expression level, as indicated. Correlation coefficients of H3K27me3 signal and gene expression decreased when window size increases, however, correlation between H3K4me3 signal and gene expression increases with window size. So +/- 300 bp around TSS was selected as the optimal window size.

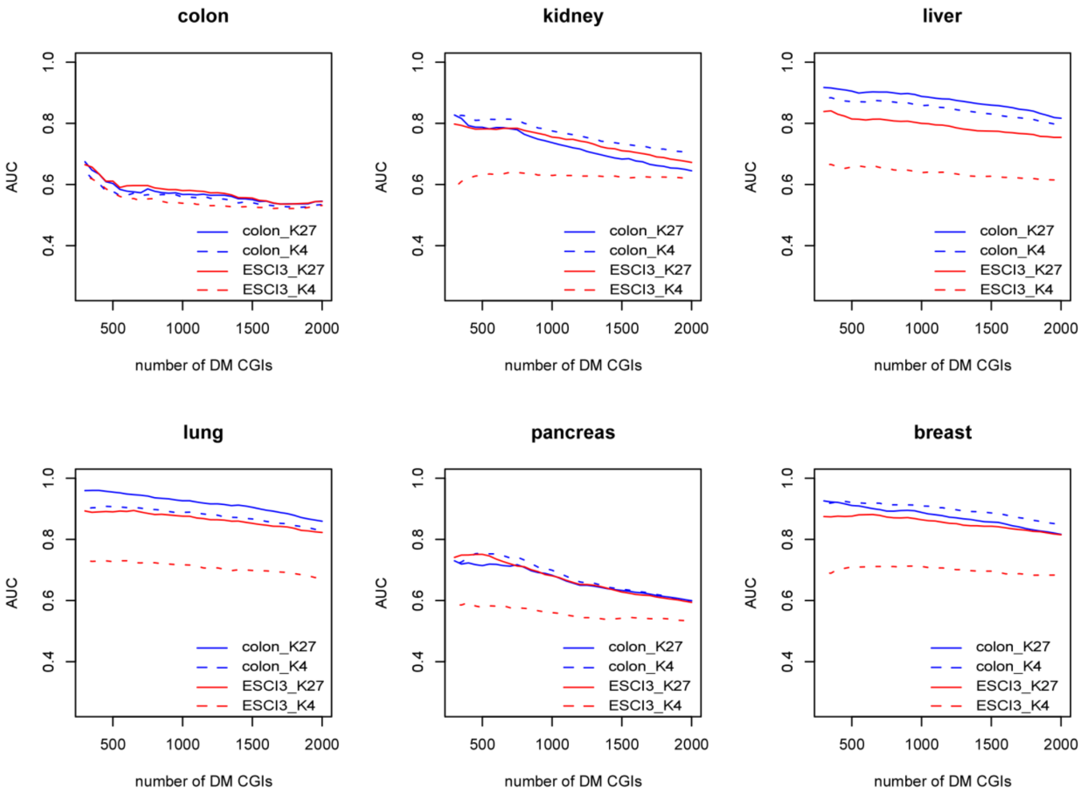

Supplementary Figure S8. Prediction accuracy of H3K4me3 and H3K27me3 marks. Gene methylation in each cancer was predicted using a binary prediction model with H3K4me3/H3K27me3 histone signal measured in normal tissues or hESCs. The AUCs of each model are shown. X-axis shows the numbers of genes which were defined as differentially methylated, and the y-axis shows the corresponding AUC of each histone mark. For most tissue types the AUCs of H3K4me3 and H3K27me3 signals derived from normal tissues of the same cell type were higher than that from hESCs, this was confirmed by a paired Wilcoxon test (p-value=0.0007 one-tailed).

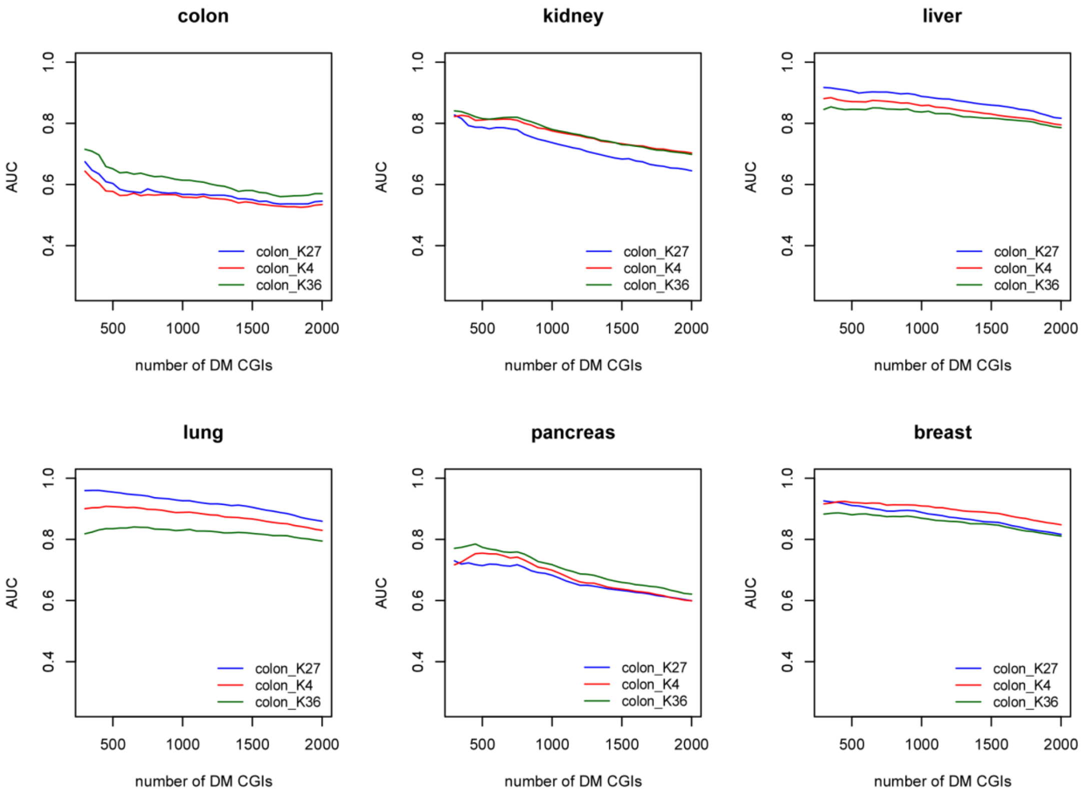

Supplementary Figure S9. Prediction of promoter DNA hypermethylation. The ability of three histone marks measured in normal tissues to predict promoter DNA hypermethylation was compared with different numbers of genes defined as differentially methylated. Y-axis shows the AUC. The H3K4me3 (red line) and H3K27me3 (blue line) show a marginally better performance than H3K36me3 (green line).

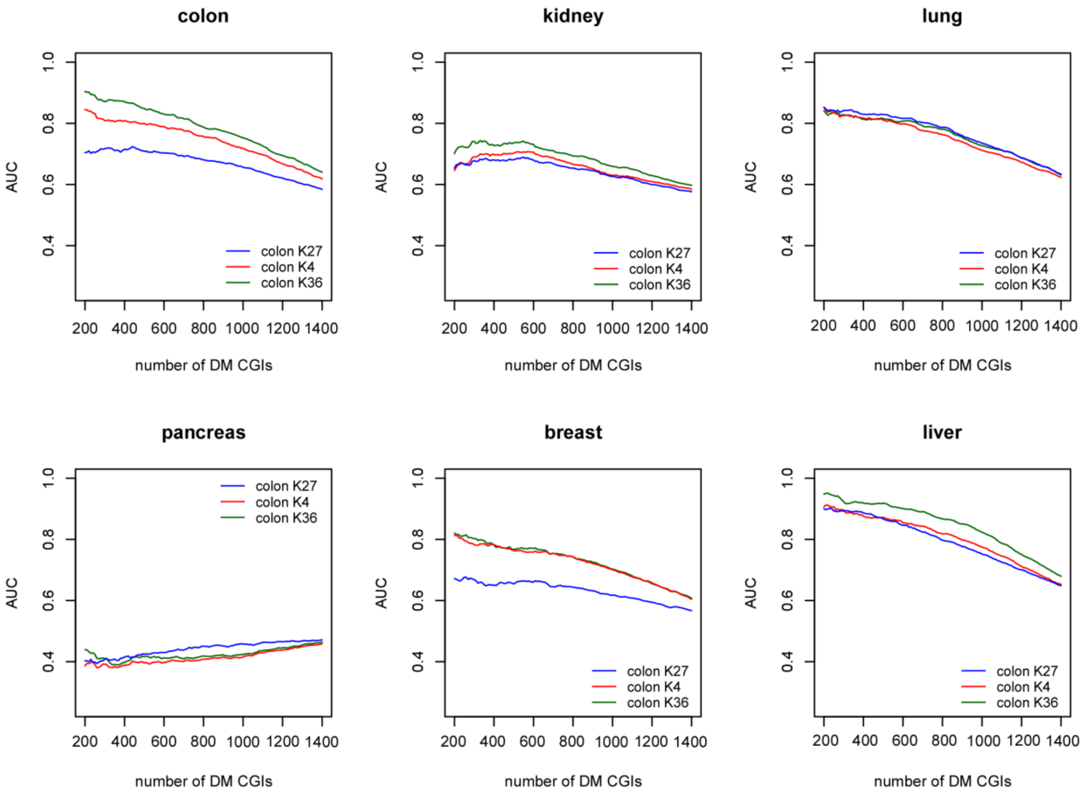

Supplementary Figure S10. Prediction of gene body DNA hypomethylation in cancer. The ability of three histone marks measured in normal tissues to predict gene body DNA hypomethylation was compared with different numbers of genes defined as differentially methylated. The H3K36me3 (green line) performed marginally better than H3K4me3 (red line) and H3K27me3 (blue line).

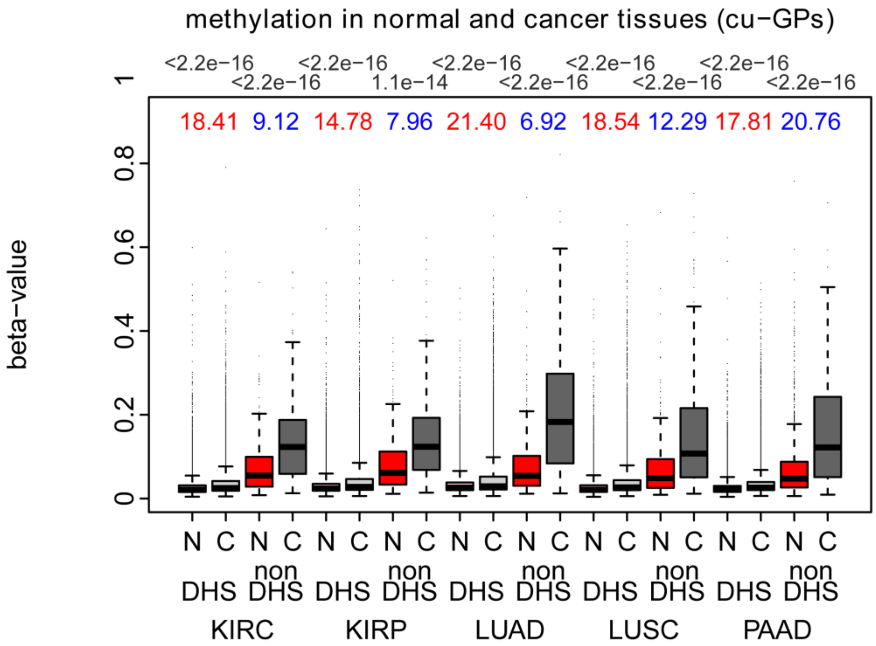

Supplementary Figure S11. DNA methylation changes of cu-GPs in cancer as a function of DHS status. Boxplots of DNA mthylation beta-values of cu-GPs, stratified according to normal/cancer tissue and whether in a DHS or non-DHS region, where DHS status is determined in the corresponding normal cell-type. DHS data was available for three normal tissues (lung, kidney, pancreas) and hence there were a total of 5 cancer types (KIRC, KIRP, LUAD, LUSC, PAAD). Above boxplots, we give the t-statistics between normal (N) and cancer (C). Red labels the t-statistics when restricted to DHS regions, blue labels t-statistics when restricted to non-DHS regions. Above the plot we give the corresponding t-test P-values.

SUPPLEMENTARY TABLE:

	CGIs in Nejman's background set	CGIs not in Nejman's background set
CGIs in our background set	5460	153
CGIs not in our background	130	164

OR=44.87
Fisher's exact test
p-value<2.2E-16

Supplementary Table S2. Agreement between our and Nejman's background (constitutively

unmethylated) CGI sets.
